# Flax (*Linum usitatissimum* L.) Fibers for Composite Reinforcement: Exploring the Link Between Plant Growth, Cell Walls Development, and Fiber Properties

**DOI:** 10.3389/fpls.2019.00411

**Published:** 2019-04-03

**Authors:** Camille Goudenhooft, Alain Bourmaud, Christophe Baley

**Affiliations:** IRDL, UMR CNRS 6027, Université de Bretagne Sud, Lorient, France

**Keywords:** biomechanics, cell wall, fiber crops, flax fiber, growth conditions, plant development, breeding

## Abstract

Due to the combination of high mechanical performances and plant-based origin, flax fibers are interesting reinforcement for environmentally friendly composite materials. An increasing amount of research articles and reviews focuses on the processing and properties of flax-based products, without taking into account the original key role of flax fibers, namely, reinforcement elements of the flax stem (*Linum usitatissimum* L.). The ontogeny of the plant, scattering of fiber properties along the plant, or the plant growth conditions are rarely considered. Conversely, exploring the development of flax fibers and parameters influencing the plant mechanical properties (at the whole plant or fiber scale) could be an interesting way to control and/or optimize fiber performances, and to a greater extent, flax fiber-based products. The first part of the present review synthesized the general knowledge about the growth stages of flax plants and the internal organization of the stem biological tissues. Additionally, key findings regarding the development of its fibers, from elongation to thickening, are reviewed to offer a piece of explanation of the uncommon morphological properties of flax fibers. Then, the slenderness of flax is illustrated by comparison of data given in scientific research on herbaceous plants and woody ones. In the second section, a state of the art of the varietal selection of several main industrial crops is given. This section includes the different selection criteria as well as an overview of their impact on plant characteristics. A particular interest is given to the lodging resistance and the understanding of this undesired phenomenon. The third section reviews the influence of the cultural conditions, including seedling rate and its relation with the wind in a plant canopy, as well as the impact of main tropisms (namely, thigmotropism, seismotropism, and gravitropism) on the stem and fiber characteristics. This section illustrates the mechanisms of plant adaptation, and how the environment can modify the plant biomechanical properties. Finally, this review asks botanists, breeders, and farmers’ knowledge toward the selection of potential flax varieties dedicated to composite applications, through optimized fiber performances. All along the paper, both fibers morphology and mechanical properties are discussed, in constant link with their use for composite materials reinforcement.

## Introduction

Flax (*Linum usitatissimum* L.) is an industrial plant of growing interest. Since its domestication started from neolithic times about 10,000 years ago (Quillien, 2014), this plant has been cultivated for its fibers, leading to its designation of “fiber crop” or “fiber plant.” Flax fibers have been used as textile raw material, composing cords and weaving yarn and later on more fashionable garments or high-quality fabric upholstery. More recently, starting around the 1930s (de Bruyne, 1939), and supported by both mechanical performances and impressive length-to-diameter ratio of flax fibers (average diameter of 20 μm for a length of 25 mm) (van Dam and Gorshkova, 2003), their applications have been extended to more technical uses, namely, as reinforcements for composite materials for the development of more sustainable materials (Yan et al., 2014). In recent years, the popularity of plant fiber-based composites has been greatly increasing to substitute glass fibers; the advantages of plant fibers compared with glass ones are their biological origin from photosynthesis and their renewable aspect, low density, low hazard manufacturing process for human health, low abrasion of processing tools, etc. (Pickering et al., 2016). Although, flax fibers have a similar range of specific mechanical properties than glass fibers, with average elastic modulus of 52.4 GPa, strength at break of 976 MPa, and strain at break of 2.15% for a fiber density of 1.53 (Lefeuvre et al., 2014b). In the case of flax-based composite materials, the fibrous part or reinforcement consists of either technical flax fibers or short fibers, impregnated with a polymer matrix. The technical fibers are composed of fiber bundles (being more or less individualized; Bensadoun et al., 2017) in a random aligned non-woven form (Merotte et al., 2016), unidirectional plies (Lefeuvre et al., 2015), or as more complex fabrics (Robitaille and Gauvin, 1999). Short fibers of a few millimeters in length and well individualized can be used to process biocomposites too, through extrusion or injection routes (Doumbia et al., 2015). The polymer matrix holds the reinforcement; it can be a thermoplastic polymer (Le Duigou et al., 2008, 2016; Dickson et al., 2014) but also a thermoset resin (Joffe et al., 2005; Åkesson et al., 2011; Mahboob et al., 2017). Finally, the development of flax-based composites is a continuously expanding research area, whether at the reinforcement scale, at the matrix scale or regarding the optimization of the reinforcement/matrix interface, and is the subject of an increasing number of papers including reviews (Summerscales et al., 2010; Ku et al., 2011; Yan et al., 2014; Pickering et al., 2016).

Thus, even though flax is the earliest domesticated plant (Allaby et al., 2005), its fibers are used in the constitution of a large range of materials, from everyday clothing to technical composites. These numerous existing applications and the constant development toward more innovative materials make flax a plant of growing industrial interest. Hence, flax is the subject of intensive works of research in the field of material science, with the aim of improving different criteria depending on whether fibers are attended for textiles or composite materials. However, much less studies focus on flax as a living plant to investigate the relationships between the properties of flax fibers and the development of the plants from which they are obtained. Among the different plants cultivated by man for fiber production, flax has many particularities that make it a unique model of development and architecture (Mikshina et al., 2013). The control of its growth allows its fibers to reach an optimized maturity and exceptional characteristics in terms of mechanical morphology and performances (Gorshkova et al., 1996; Baley and Bourmaud, 2014). Moreover, for nearly 100 years, it has been the subject of a varietal selection that has made it a plant with a strong technical potential but also a great source of remuneration for farmers (Jankauskiene, 2014). The varietal selection is most probably the origin of the uncommon slenderness of flax, which allows to consider flax plant as a model of stability and a source of bioinspiration (Baley et al., 2018). Like any plant, it reacts to external stresses and its development and stem architecture can be influenced by thigmomorphogenesis, gravitropism, as well as environmental or cultivation conditions (Moulia et al., 2006).

The present work proposes a review of the literature to highlight possible links between the characteristics of flax plants and their possible impact on the fibers performances. In the first section, the main growth stages of flax plants and the internal organization of the stem biological tissues are investigated. In addition, the development of flax fibers, from their elongation to their thickening, is reviewed. The slenderness of flax is then highlighted through a comparison with herbaceous plants and trees. Second, the selection of new flax varieties and related selection criteria are explored. This second section includes an overview of the influence of the varietal selection on plant characteristics, together with a more extensive review of the changes inducing an improved lodging resistance. Finally, the last section explores the influence of the cultural conditions, more particularly of the seedling, on the stem and fiber characteristics. The impact of environmental conditions, such as the wind flow in a plant canopy and main tropisms, is reviewed.

## Growth of the Flax Plant and Development of Its Fibers

### Growth of the Plant

The ideal climate for flax cultivation is in temperate and humid regions where the daily temperature does not exceed 30°C and providing about 700 mm of annual rainfall; for instance, the temperate and maritime areas of Belgium, Netherlands, or France in coastal Western Europe are highly suitable locations for the flax cultivation (Sultana, 1992). The cultivation of flax starts with sowing, usually when the upper layer of soil reaches about 7–9°C, i.e., for example, between 15th of March and 15th of April in France (Sultana, 1983). Afterward, the growth stages of flax are divided into four main steps, like most crops: G (or emergence of the plant), VS, F and SF, and finally S (Lancashire et al., 1991; Mediavilla et al., 1998). Germination usually starts around 5–10 days after sowing (Gorshkova et al., 1996; ARVALIS - Institut du végétal, 2014), and is characterized by the apparition of two fully developed cotyledons (Paul-Victor et al., 2017) which justify its classification as a dicotyledonous plant (Edwards et al., 1997). The VS starts quite slowly, the flax plant reaching 15 cm after about 15–20 days after G (Gorshkova et al., 2003b; ARVALIS - Institut du végétal, 2014). This slow development is followed by a period of fast growth that takes place during about 15–20 days (Gorshkova et al., 2003b; ARVALIS - Institut du végétal, 2014). The flax plant is able to elongate several centimeters per day during fast growth (Gorshkova et al., 1996; Heller et al., 2015), reaching 80–90 cm over this 2-week period of fast development (Gorshkova et al., 1996; ARVALIS - Institut du végétal, 2014). Then, plant growth slows down and finalizes during F, and the plant reaches a final length of about 1 m (Gorshkova et al., 2003b). Flowering starts about 50 days after G; it lasts about 15 days for a whole field, even though a single flower lasts only 1 day (Bert, 2013). Seeds, contained inside capsules, are formed from 15 days after F; their full maturity is reached “late ripening,” happening after 5–6 weeks after F (Tiver and Williams, 1943). Finally, like for all plants, S takes place (Lancashire et al., 1991). However, in the case of industrial flax plants, neither SM nor the S are reached. In fact, plants are pulled out at FM, i.e., about 40 days after F or “yellow ripening,” phase that succeeds the “green ripening” (or “early-ripening”) (Gorshkova et al., 1996). Finally, the cultivation of flax, from sowing to FM, takes about 100–120 days.

In addition to farmers’ experience, the different stages of intervention during the flax growing cycle are also determined taking into account the cumulative temperature received by the plant after sowing, also called cumulative growing degree-day (Prentice et al., 1992). On day *n*, the cumulative growing degree-day formula (*GDD_n_*) is calculated as:

$$GDD_{n}=\sum_{i=1}^{n}\frac{T_{max,i}+T_{min,i}}{2}-T_{base}$$

where *T_max,i_* is the maximal daily temperature, *T_min,i_* the minimal daily temperature on the day *i* (with *i* equals to 1 on the day of sowing), and *T_base_* the base temperature. For flax, the base temperature is equal to 5°C, as it is considered to be the zero vegetation for this plant (i.e., the temperature below which no growth occurs) (Bert, 2013), whereas it is usually 10°C for most crops. In the case of an average daily temperature being lower than the base temperature, *GDD_i_* is considered equal to 0.

**FIGURE 1:**
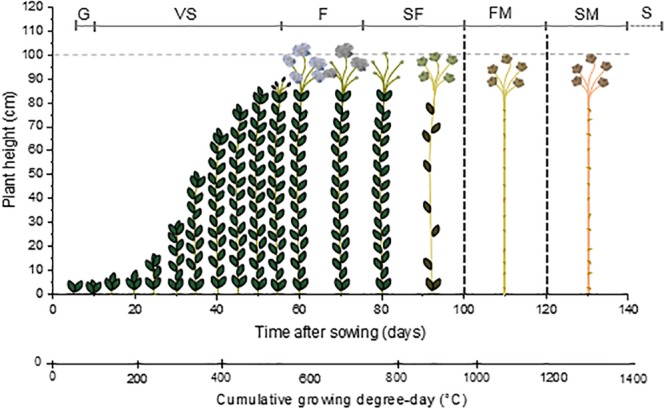
Scheme illustrating the growth of flax starting from the sowing day, according to the time after sowing and corresponding cumulative growing degree-day (GDD). *G*: germination; *VS*: vegetative stage; *F*: flowering; *SF*: seed formation; *FM*: fiber maturity; *SM*: seed maturity; and *S*: senescence.

By taking into account this method of calculation, the G of flax happens when the cumulative GDD reaches about 50°C. Flowering takes place for a cumulative GDD of 550°C whereas the capsules are formed when a higher cumulative GDD is reached, namely, around 650–700°C. Finally, fibers are considered mature when the cumulative GDD reaches from 950 to 1100°C. The seeds would be mature around 1150°C but plants are actually pulled out at FM (ARVALIS - Institut du végétal, 2014). The scheme visible in Figure 1 illustrates the path of flax growth according to the cumulative GDD. Under different growth conditions (such as temperature or amount of rainfall) (Lefeuvre et al., 2014b; Du et al., 2015) or different cultivation methods (such as sowing density) (Bourmaud et al., 2016), the growth pattern of flax is modified. In summary, Figure 1 only gives a general indication of the flax growth stages.

### Internal Organization of Flax Biological Tissues

Flax is, like every plant, composed of different body parts known as plant organs; the vegetative organs can be reduced to the stem, the leaf and the root, each having particular functions (Arber, 2012). The plant organs form a continuous structure because they have a common origin. The present section as well as the whole paper will focus only on the stem, which is the key organ of the flax fiber ontogeny.

The stem has two main functions: conduction and support (Gartner, 1995). To perform its vital functions, the stem is itself composed of different elements, i.e., several types of tissues made of grouping of different cell types distinguishable in position, structure, origin, or developmental phase (Romberger et al., 1993). The tissues composing the stem can be separated in three basic tissue systems: epidermal, vascular, or ground (Romberger et al., 1993). Each of them is initiated by differentiating meristems, i.e., by regions where non-differentiated cells have the ability to divide, in order to produce additional cells that will differentiate (Evert and Eichorn, 2013), such as fibers. Meristems involved in the longitudinal growth of the stem are called primary meristems and are located at the top of the shoot (and roots) (Meicenheimer, 1992); they are responsible of the primary growth that leads to primary tissues (Schweingruber et al., 2006). For example, procambium is a primary meristem that is responsible for the primary vascular system (Esau, 1942). On the other hand, the secondary growth, leading to secondary tissues, originates from lateral meristems. For instance, the vascular cambium is a lateral meristem responsible for the secondary vascular tissues. This secondary growth is responsible for the thickening of the stem, rather limited and essentially attributed to the secondary xylem in the case of flax (Schweingruber et al., 2006). Thus, flax is an herbaceous plant, a category which refers to plants expressing either a lack of or a poor organ secondary growth. The arrangement of the tissues within the stem defines and enables the functionality of this organ (Evert, 2006). However, it is difficult to separate cells and tissues into clear categories, as the whole stem system comes from the interaction of each of them. The information given in Table 1 presents a general classification based on literature about plant structures, that slightly differs between authors (Romberger et al., 1993; Gartner, 1995; van Dam and Gorshkova, 2003; Evert, 2006; Schweingruber et al., 2006; Evert and Eichorn, 2013); in addition, this present classification focuses on main cell types found in flax stems so is not exhaustive.

**Table 1:** Main tissues and cell types composing flax stems.

Basic tissue system	Tissue	Cell type	Location	Main functions
Dermal	Epidermis		Outermost layer	Protection
Ground	Parenchyma	Parenchyma	Through the stem body	Metabolism
	Sclerenchyma	Fiber	Bundles in phloem	Mechanical support
Vascular	Phloem	Sieve-tube element	Phloem	Food/elaborated sap conduction
		Companion cell	Phloem	Delivery of substances to the sieve-tube element
	Xylem	Vessel element	Xylem	Water and raw sap conduction
				Mechanical support
		Tracheid	Xylem	Water and raw sap conduction
				Mechanical support

**FIGURE 2:**
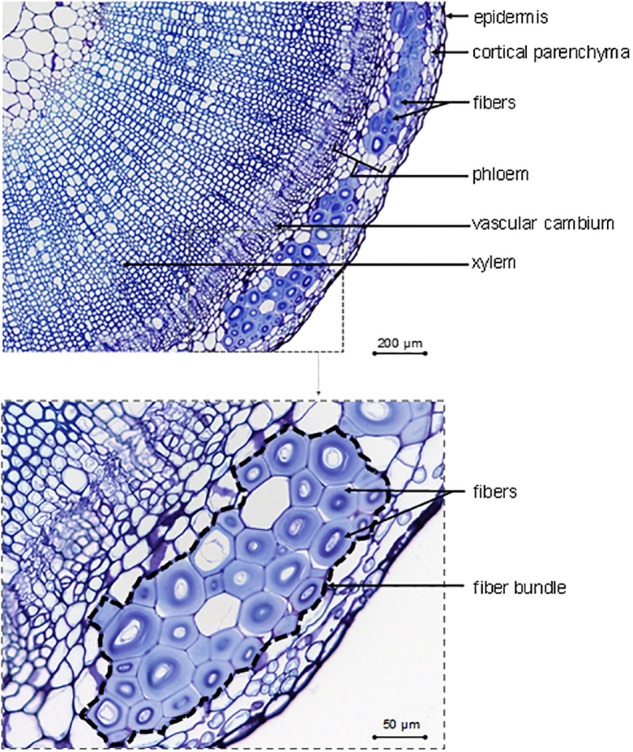
Description of identifiable tissues and cells on the transverse cross-section in the bottom part of a growing flax stained with toluidine blue.

As a complement to Table 1, the main tissues or cells visible on a transverse cross-section are identified in Figure 2, with fibers gathered in bundles (up to 40 cells per bundle; McDougall et al., 1993) present at the periphery of the section. Figure 2 also shows imperfections, such as a heterogeneous fiber thickening within the same bundle for a given cross-section, which is part of this natural composite structure. Finally, flax fibers, occurring in bundles, are elements of the sclerenchymatous tissue located in the primary phloem (Esau, 1943), which has led to the terms “bast fibers” (Aldaba, 1927; Esau, 1943; Bergfjord and Holst, 2010), “primary phloem fibers” (Evert, 2006; Gorshkova and Morvan, 2006; Gorshkov et al., 2017), or “pericyclic fibers” (Tiver, 1942; Catling and Grayson, 1982) sometimes found in literature. Contrary to some other fiber plants, flax generates only primary fibers, i.e., fibers originate exclusively from primary growth (van Dam and Gorshkova, 2003). For example, hemp (*Cannabis sativa* L.) has both primary and secondary fibers, whose latter are subjected to specific studies (Snegireva et al., 2015; Bourmaud et al., 2017; Fernandez-Tendero et al., 2017). However, the formation of secondary fibers will not be detailed in the present work for the reasons mentioned above.

### Cell Growth of Flax Fibers

In terms of plant biology, a fiber is an individual sclerenchyma cell that provides mechanical support to the plant. In addition, a fiber is characterized by an extreme length along with a length-to-diameter ratio higher than 1000 (van Dam and Gorshkova, 2003). Besides, this type of cell exhibits an impressively thick cell wall, as this latter can reach a ten micrometers in thickness, i.e., around 10 times thicker than most other cell types (Mikshina et al., 2013). Additional characteristics of a fiber are tapered ends and spindle-like shape (Gorshkova et al., 2012). Those remarkable properties, and more interestingly the intensified elongation and cell wall thickening, are verified by flax fibers, making them an appropriate model of plant cell growth.

**FIGURE 3:**
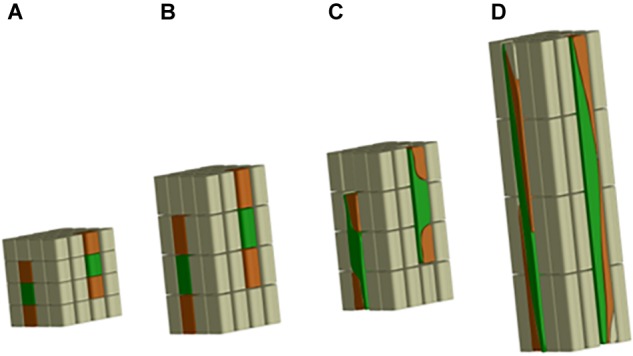
Scheme illustrating the fiber elongation, with fibers represented in green. **(A)** First phase of the coordinated growth; **(B)** more advanced step of the coordinated growth; **(C)** beginning of the intrusive growth with fibers having “knees” on both ends; **(D)** more advanced phase of the intrusive growth, with fibers becoming a much longer structure than the neighboring cells and showing tapered ends.

**FIGURE 4:**
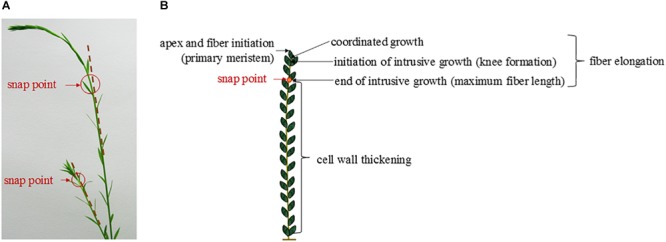
**(A)** Pictures of two flax stems of different heights having a different snap point location. **(B)** Scheme illustrating the different stages of the fiber formation along a flax stem according to the localization of the snap point.

In the case of flax fibers (referred to here as “fibers”), the cells are initiated by primary growth, from the apical meristem (Anderson, 1927; Ageeva et al., 2005). The fiber initiation is then followed by two main stages: elongation and cell wall thickening (Roach and Deyholos, 2007). The stage of elongation is particularly outstanding in the case of flax fibers, as their final length can reach up to 65 mm with average values of 25 mm (Gorshkova et al., 2003a). These extreme values could be explained by the two-step process of elongation: coordinated growth then intrusive growth (McDougall et al., 1993). Actually, the elongation process of flax fibers starts with coordinated growth, i.e., fibers elongate synchronously with neighboring cells (Ageeva et al., 2005). Coordinated growth starts the fiber elongation from cell formation up to first millimeters from the apex, and it occurs for all types of cells of the upper region of the stem (van Dam and Gorshkova, 2003). However, diverse sizes between cell types can result from coordinated growth, due to different cell division frequency and timing of division cessation (Snegireva et al., 2010). The coordinated growth of a fiber leads to multinucleate cells and lasts only for several hours, at the end of which the cell reaches averages of 100 μm in length for about 5 μm in diameter (Ageeva et al., 2005; Snegireva et al., 2010). Fiber elongation continues with intrusive growth, when the rate of fiber longitudinal growth exceeds that of neighboring cells still growing by symplastic growth; in this case, the fiber cell forces its way in between other cells and both of its ends form a “knee” being the characteristic of the beginning of the intrusive growth (Figure 3) (McDougall et al., 1993; Ageeva et al., 2005). The shapes of knee disappear with the advance of the intrusion, and the apparition of the spindle-like shape with tapered ends happens as the fiber elongates (Ageeva et al., 2005). In addition, the intrusive growth is realized by diffuse growth, i.e., the whole surface of the fiber is elongating (Gorshkova et al., 2003b) even though the diffuse elongation can have different rates over the fiber length which explains the formation of knees and tapered ends (Ageeva et al., 2005). The intrusive growth lasts longer than coordinated growth of the fibers, namely, several days during which the elongation ranges about 1–2 cm per day (Gorshkova et al., 2003b). Thus, intrusive growth happens starting below the first millimeters to the snap point region, and leads to fibers reaching several centimeters in length (average of 25 mm previously mentioned) for an average diameter of 20 μm (van Dam and Gorshkova, 2003). The so-called “snap point” is defined as the crucial point past which no fiber elongation occurs, i.e., the length of a fiber reaches its maximum at the level of the snap point (Gorshkova et al., 1996, 2003b) (Figure 4). In addition, as intrusive growth no longer occurs below the snap point, the number of fibers on a transverse cross-section at a given stem height does not increase anymore at or below the snap point (Gorshkova et al., 2003b). As the plant develops, the snap point moves along the stem; it is identifiable from the beginning of the fast growth until the plant ends its growth, and located up to 10 cm below the apex (Gorshkova et al., 2003b). Furthermore, the snap point is simply identified as “the point where a difference in load is needed to break the stem in tensile” (Gorshkova et al., 2003b), due to a major change into fiber thickening and consequently stem bending stiffness, with much softer stem above the snap point. Impressively, the softer parts sometimes exhibit an almost vertical position of the top part of the plant with its own weight and wind (Figure 4A). This phenomenon of changes in stiffness is directly related to the second stage of fiber formation, namely, cell wall thickening, happening essentially below the snap point (Gorshkova et al., 2003b). Cell wall thickening is a much longer process than cell elongation, as it lasts weeks until plant maturity, i.e., it can take up to 60 days (Gorshkova et al., 2003b; Ageeva et al., 2005). Thus, this stage is a long and complex process that is detailed in the dedicated following subsection.

### Cell Wall Thickening

Due to the impressive thickness of the flax fiber cell wall compared to other plant species (flax fiber cell walls can reach more than 10 μm whereas most cell walls are few microns thick) (Mikshina et al., 2013), the process of fiber thickening has been subjected to many studies. This process results in a uniform cell wall deposition along the fiber length but several cell wall layers, whose deposition is described hereafter (Snegireva et al., 2010).

**FIGURE 5:**
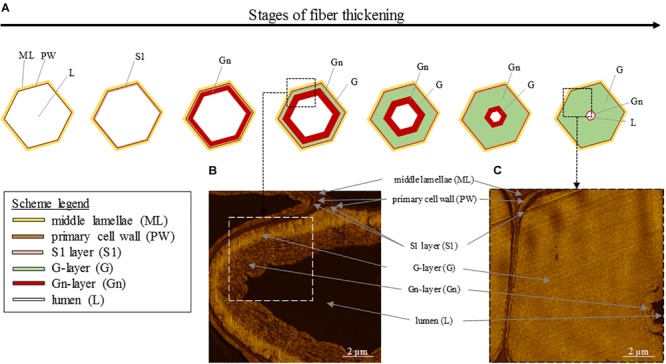
**(A)** Scheme illustrating the different stages of the fiber thickening, starting from a cell having only a primary cell wall (PW) and ending with a fiber having a thick G-layer, a small lumen and a possibly remaining thin Gn-layer. **(B)** AFM image of an intermediate stage of cell wall thickening showing the G-layer and the innermost newest Gn-layer. **(C)** AFM image of a final stage of cell wall thickening showing the thick G-layer and the innermost residual Gn-layer.

At its formation, flax fiber cell, similarly as other plant cells, is constituted by a PW and stuck with other cell through the surrounding ML (Gorshkova et al., 1996; Cosgrove and Jarvis, 2012). PW of flax fibers is a very thin and extensible structure able to withstand the impressive fiber elongation. In addition, it has the ability to withstand the deposition of the newest cell wall layers (Gorshkova et al., 2018). This foremost additional layer to be deposited inside the cell on PW is the so-called S1 layer, the first and very thin layer of the SW (Domenges and Charlet, 2010; Rihouey et al., 2017). The further deposited layers responsible of the extreme fiber thickening, and more specifically their denomination, are the subject of debates between authors. Based on a recent article focusing on mature fibers (Rihouey et al., 2017), it is possible to describe the next additional layer as the major thick layer denominated as S2 SW layer or preferentially G-layer because of its specific properties. Namely, the G-layer is fiber-specific and has a pronounced content of cellulose (up to 90%) with a high crystallinity, a low angle of cellulose microfibrils being almost parallel to the fiber longitudinal axis (with a MFA reaching 8–10°), and is also characterized by the absence of both lignin and xylan, its high water content, its impressive porosity, as well as contractile properties (that will be detailed further) (Mikshina et al., 2013). This remarkable cell wall layer that is the G-layer has led to the term “G-fiber” sometimes used to name the fibers having such a layer (Bowling and Vaughn, 2008; Roussel and Clair, 2015; Ibragimova et al., 2017). Lastly, the innermost thin deposited layer is defined as S3 or Gn-layer (Hearle, 1963; Rihouey et al., 2017). The organization of the different cell wall layers and main stages of fiber thickening are illustrated in Figure 5 (AFM protocol from literature; Goudenhooft et al., 2018). In addition, the Gn-layer is usually hardly visible at the end of the thickening reached FM (Figure 5); nevertheless, this Gn-layer can remain in cases of exogenous accidents such as drought or lodging during plant growth (Gorshkova et al., 2010b). Thus, this latter layer is defined as a transient layer. In fact, the newest Gn-layer progressively transforms into the mature G-layer over cell wall thickening, gradually increasing the thickness of the G-layer (Gorshkova et al., 2018). Gn- and G-layers differ in appearance, namely, the Gn-layer exhibits a loosen structure whereas the G-layer shows a much more compact and homogeneous configuration (Gorshkova et al., 2004), leading to a greater nanoindentation modulus of the G-layer (Goudenhooft et al., 2018). The differences that can be extended to other cell wall layers are explained by their composition as well as the microfibrils arrangement within layers.

### Cell Wall Composition and Organization

**FIGURE 6:**
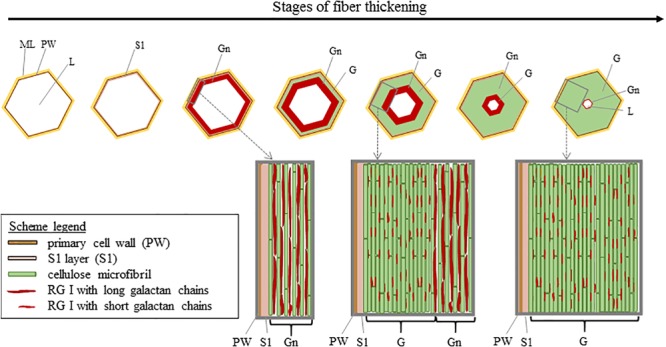
Scheme illustrating the cell wall thickening in relation to the remodeling of the Gn-layer. Cellulose microfibrils are originally separated by long galactan chains present in nascent RG I [5]. The long galactan chains are then trimmed off which leads to the G-layer with compact cellulose microfibrils. Scheme inspired from Goudenhooft et al. (2018).

The composition and organization of flax cell wall constituents are the subject of numerous debates as they are different between authors (McDougall, 1993; Morvan et al., 2003; Gorshkova et al., 2010b). These characteristics are of great interest as they can be correlated to the mechanical performances of flax fibers (Lefeuvre et al., 2014a). Table 2 attempts to give a general overview of the cell wall architecture, considering that this latter is influenced by several parameters (such as the flax selected variety, growth conditions, cultural practices, etc.) (Milthorpe, 1945; Alix et al., 2009) and the disagreements between sources. In addition, the term “hemicelluloses” used in Table 2 is sometimes replaced by “neutral polysaccharides” in literature, to echo back the “pectic” polysaccharides that are pectins (Rihouey et al., 2017). One can notice that the approximate composition of the Gn-layer is not well detailed in literature. However, the composition of the Gn-layer is expected to be quite similar to the G-layer one, as this latter is supposed to be a remodeling of the Gn-layer (Gorshkova et al., 2018). G- and Gn-layers, due to their high cellulose content, have a great affinity with toluidine blue (Figure 2), which facilitates the fiber microscopic identification. The mentioned remodeling consists of the modification or trimming of the RG I having long galactan chains by specific β-(1;4)-galactosidase (Roach et al., 2011). The shortening of the RG I galactan chains enables stronger lateral interaction of cellulose microfibrils; thus, it leads to transform the heterogeneous loosen Gn-layer enriched in long chains of galactan into the homogeneous and mature G-layer composed of shortened and entrapped galactan chains (Figure 6) (Gorshkova et al., 2018).

**Table 2:** Characteristics and approximate composition of the different cell wall layers of flax fibers.

Cell wall layer	Average thickness	Microfibrils orientation	Approximate composition
PW	0.2 μm (Bos and Donald, 1999)	disperse orientation, preferentially 0° (Cosgrove and Jarvis, 2012; Rihouey et al., 2017)	∼25–40% Cellulose ∼30% Hemicelluloses (mainly xyloglucan and lesser amounts of arabinoxylan) ∼30% Pectins (mainly homogalacturonan; possibly rhamnogalacturonan (RG) I; RG II and arabinogalactan) (Carpita and Gibeaut, 1993; Andème-Onzighi et al., 2000; His et al., 2001; Morvan et al., 2003; Cosgrove and Jarvis, 2012; Mellerowicz and Gorshkova, 2012)
S1	0.5 μm (Domenges and Charlet, 2010; Bourmaud et al., 2013)	60–80° (Barnett and Bonham, 2004)	∼30–50% Cellulose ∼30% Hemicelluloses (xylan, xyloglucan) ∼5% Pectins (homogalacturonan and RG I) ∼10–20% Lignin (Gorshkova et al., 2010a; Cosgrove and Jarvis, 2012; Mellerowicz and Gorshkova, 2012; Rihouey et al., 2017)
G	Up to 15 μm or 90% of the total cell wall area at maturity (Morvan et al., 2003; Gorshkova et al., 2018)	8–10° (Bledzki and Gassa, 1999; Bourmaud et al., 2013)	∼75–90% Cellulose ∼15–20% Hemicelluloses (glucomanan) ∼5–10% Pectins (RG I) (His et al., 2001; Morvan et al., 2003; Mellerowicz and Gorshkova, 2012; Rihouey et al., 2017; Gorshkova et al., 2018)
Gn	0.5–1 μm through thickening (Bourmaud et al., 2013; Arnould et al., 2017)	Loosely packed as a heterogeneous structure (Roach et al., 2011)	Cellulose Hemicelluloses (glucomanan) Pectins [nascent RG I (i.e., long galactan chains)] (Gorshkova et al., 2010a, 2018; Roach et al., 2011; Rihouey et al., 2017)

### The Slenderness of the Flax Stem: An Incredibly Slender Structure in the Plant Kingdom

**FIGURE 7:**
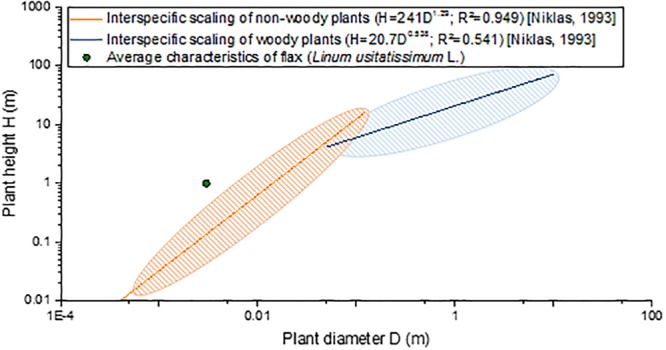
Interspecific scaling of plant height (*H*) as a function of the plant basal diameter (*D*) of flax in comparison with scaling formulas and maximal dispersion of non-woody plants (190 species) and woody plants (420 species) from data proposed by Niklas (1993b).

“Slenderness” is defined as the ratio between the height of a column to its least radius of gyration (Niklas, 1992), a definition that can be applied to engineered structures but to plants as well. More commonly for plants, slenderness is defined as the ratio between the maximal height and the basal diameter of the stem (Romberger et al., 1993; Niklas, 1994). Based on literature, flax stems have no identified challenger in terms of slenderness, compared with plants exhibiting the same range of diameter (namely, herbaceous plants, also called non-woody plants). In fact, taking an average total height of 1 m and a slightly overestimated average basal diameter of 0.003 m (van Dam and Gorshkova, 2003; Gibaud et al., 2015), flax possesses a greater slenderness than herbaceous stems, for example, in comparison with 190 other non-woody species studied by Niklas (1993b) (Figure 7). The comparison has to be interpreted carefully, as flax is a plant cultivated under an optimal context, whereas most other plants are wild ones. Moreover, despite being an herbaceous plant, flax remarkably appears to present a scaling relationship more closely resembling woody plants (including trees) rather than non-woody ones (Figure 7). Thus, the slenderness factor highlights geometric aspects of the plant (namely, its length and diameter), making flax an incredibly slender structure among the biological world. This interesting characteristic of flax can be attributed to the internal organization and properties of its composing tissues, but the varietal selection work is most likely the main cause that has led to incredibly slender flax plants. In fact, although the varietal selection of food crops has been focusing on breeding dwarf cultivars to decrease their lodging vulnerability (Crook and Ennos, 1994; Oladokun and Ennos, 2006), the flax varietal selection has never intended to reduce the plant height. Indeed, on the contrary, reasonably high plants are desired to provide higher yield, as fibers are extracted from the stem.

Based on the vocabulary and development stages of flax stems and fibers previously presented, the following section is mainly dedicated to review the literature about the work of varietal selection of industrial crops and more particularly of flax.

## Evolution of Industrial Crops and Varietal Selection

**FIGURE 8:**
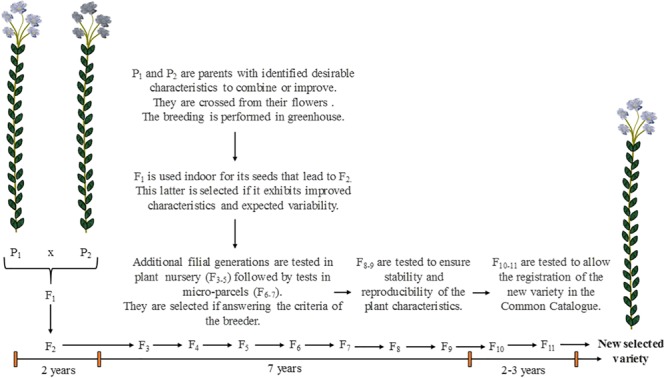
Scheme illustrating the main steps of the varietal selection in the case of flax, from the parents P_1_ and P_2_ to the numerous filial generations F_i_ to achieve the obtainment of a new selected variety. Scheme inspired from Bert (2013) and Evert and Eichorn (2013).

The selection of crops, in the present paper also called “varietal selection,” is a work of great importance to achieve plant cultivation for industrial purposes. The varietal selection is the process or work through which breeders intent to change the regular characteristics of a domesticated plant in order to promote it with one or several desirable properties (Evert and Eichorn, 2013). In few words, it consists of breeding existing varieties having wanted properties to provide a new selected variety (also called “cultivar”) that will exhibit the advantages of both parents (Figure 8). Thus, this selection is an artificial process that would not occur naturally. For flax, being a self-pollinating plant, crossing is performed manually from the flowers by breeders. More than 10 years are required to provide a genetically fixed variety and for its proper multiplication (Bert, 2013). The detailed principle of the varietal selection and how it affects the plant genetics will not be discussed in the present review, that rather aims at explaining the criteria of selection and their influence on the plant characteristics.

### New Varieties of Major Industrial Cropsand Selection Criteria

Among industrial plants, cereals such as rice (*Oryza sativa*), wheat (*Triticum aestivum*), rye (*Secale cereale*), barley (*Hordeum vulgare*), or maize (*Zea mays*) have been domesticated then selected over the years. The major goals of their selection are for instance higher grain yields, larger grains, thicker straws, or seeds that separate easier from the chaff. Additionally, fiber crops are also subjects of the varietal selection. One major example of a fiber crop selected for its fibers is hemp (*Cannabis* sativa L.) (Ranalli, 2004). In the case of hemp, the use of this fiber crop used as a raw material for the manufacture of rope, fabric or paper goes back to 6000 years ago (Schultes, 1970). Its varietal selection started about 200 years ago, and nowadays less than 50 European fiber cultivars are registered (Ranalli, 2004). In the same way as for food crops, one of the major goals of the varietal selection of hemp is to increase the plant yields, mainly in terms of fiber content of the plant but also in seeds and woody core (van der Werf et al., 1996). Contrary to flax, hemp has the ability to produce secondary fibers (Bourmaud et al., 2017). The number of secondary fibers should be limited, as these short and highly lignified fibers bring scattering in performances and are not desirable for most applications (van der Werf et al., 1994; Placet et al., 2014). Thus, cultivars with low secondary fiber content should be privileged by breeders. Last but not least, an additional parameter specific to hemp has to be considered regarding the varietal selection, namely, the THC content of the plant. This psychoactive substance has to be monitored in order to represent lower than the legal limit of 0.2% THC (Grassi and Ranalli, 1999; Callaway, 2008).

Hence, since the beginning of agriculture, crops have been domesticated by humans, mainly to produce food but also raw materials. Over the years, farmers have saved seeds of plants with advantageous characteristics to domesticate plants exhibiting the naturally most attractive properties. More recently, started about 200 years ago, breeders have been crossing plants and have been selecting new varieties to provide improved plants; this process is called varietal selection and could be referred to as “selective breeding” (Evert and Eichorn, 2013). The selection criteria are slightly the same from an industrial crop to another, even though some crops have additional required selection criteria, such as hemp which also has legal restrictions. However, a more recent type of breeding is currently expected to go beyond selective breeding and overcome the plateau beyond which selective breeding is not able to increase yields anymore (Peng et al., 2009; Evert and Eichorn, 2013); it is referred to as “plant biotechnology” and involves genetic engineering such as recombinant DNA technology (Wielgus et al., 2012; Evert and Eichorn, 2013). This additional type of breeding is considered beyond the scope of this work, and will not be presently discussed.

### Focus on New Flax Varieties and Related Selection Criteria

In the category of industrial crops, flax is also a major plant whose current cultivars are the result of the varietal selection. Selected for two main purposes, namely, its seeds and its fibers, a different approach was adopted by breeders according to the expected applications, leading to two distinct groups of cultivars (Heller et al., 2015). In addition, it is also known that some cultivars were selected to provide a balance between seed and fiber yields (Foster et al., 1998). The present review of the flax selection focuses on the development of fiber varieties exclusively.

From the time when agriculture was developed, i.e., about 10,000 years ago, humans have domesticated and intuitively selected the strains of flax that gave the best-looking plants providing fibers of good quality (Evert and Eichorn, 2013; Quillien, 2014). The seeds of these plants with attractive properties were kept and sowed the following year, expecting desirable traits to come again. More recently, like other industrial crops, humans have purposely been crossing and selecting flax plants to produce improved varieties. Literature suggests that current varieties of fiber flax originated from older varieties developed in Eastern Europe (Helbaek, 1959; Allaby et al., 2005). More precisely, it is possible that flax breeding started in Lithuania in 1922, after more than 4000 years of cultivation in this country (Razukas et al., 2009). Nowadays in 2018, after a century of varietal selection, this work is performed by only a few breeders; fewer than 50 fiber flax varieties, excluding any GMO one, are for instance currently registered in the Common Catalog of France and commercially used.

In 2017, seven new varieties were registered, which demonstrates the dynamism regarding the work of flax varietal selection. Moreover, older varieties are still registered in 2018, such as Marylin or Alizée, even though more recent ones were registered and deleted in the meantime; the numerous deletions confirm the challenge and investment of flax breeders to select varieties with actual improved qualities. For economic reasons, since the beginning of flax breeding and even more in today’s world facing the development of flax-based composites (Yan et al., 2014), the principal aim of textile flax breeders is to select high fiber yielding flax varieties to meet the industrial demand (Jankauskiene, 2014). A numerical example of the incredible increase of the fiber yield could be the following one: in France in 1930–1935, the average fiber yield was about 0.30–0.65 t/ha (Chevalier, 1944) whereas in 2015, an average fiber yield of 2.02 t/ha was obtained with the highest value of 3.92 t/ha for the recent cultivar Aramis in experimental fields (ARVALIS - Institut du végétal, 2016). A recent article (Goudenhooft et al., 2017) illustrates the general increase of straw and fiber yields since the beginning of the varietal selection, in the case of France. More precisely, increase of about 35 kg/ha of fibers was obtained each year between 2003 and 2013, corresponding to about +2.3% in fiber yield each year (Bert, 2013). Of course, the increase of fiber yield is attributed to the work of varietal selection, but is also due to the mechanization that appeared in parallel starting from the 1960s, as well as higher G rate (about 92%; Bourmaud et al., 2016) and a rise of scientific studies focusing on flax providing a more precise knowledge of the plant growth (Sultana, 1992; O’connor and Gusta, 1994; Montaigne, 1997). In addition, the harvested straw yield only slightly increases over the years despite mechanization, which confirms the key role of the varietal selection in the increase of fiber yield.

Furthermore, the resistance to diseases is also a selection criterion for flax breeders, as diseases can be devastating for cultures and are thus limiting factors in flax production. For instance, the resistance to the fungal pathogen fusarium wilt (*Fusarium oxysporum*) is a priority of the varietal selection of flax (Spielmeyer et al., 1998). Moreover, the powdery mildew (*Oidium lini*) is also a major flax disease (Rashid and Duguid, 2005), as well as the different races of flax rust (Rashid, 1991) or flax scorch (*Phytium megalacanthum*) (Wiersema, 1955), and the resistance against them is a desired characteristic for new cultivars and challenges for breeders.

**FIGURE 9:**
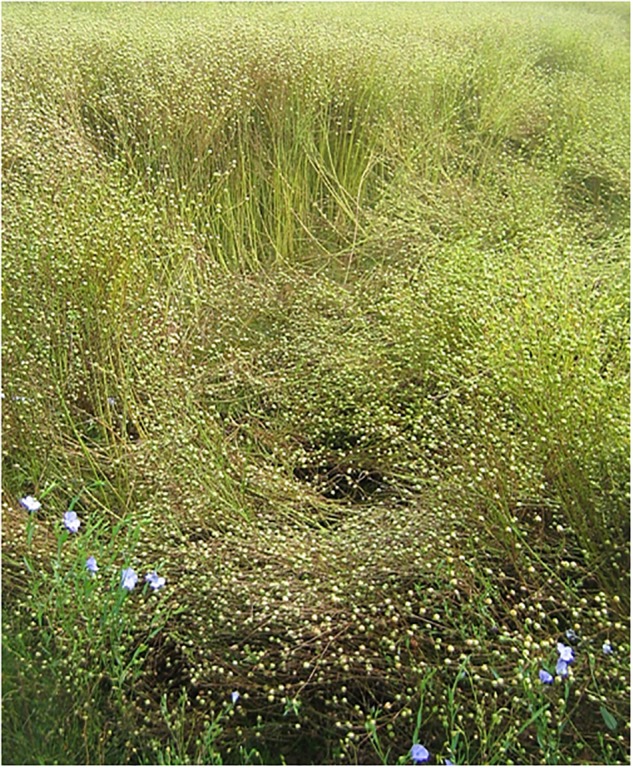
Lodged flax plants in a field at the end of the F stage.

Finally, another criterion is the priority for flax breeders, namely, the lodging resistance. The phenomenon of flax lodging is illustrated in Figure 9. This undesired event is also a major selection criterion for rice or wheat (Crook and Ennos, 1994; Oladokun and Ennos, 2006). Lodging generally happens after rainfalls and wind (Vera et al., 2012). For the food crops, the main solution proposed to prevent lodging is the selection of dwarf varieties, i.e., shorter stems (Berry et al., 2006, 2007). This is indeed a suitable solution for those crops whose grains are located in the ears, the stem having only a supporting role. For flax, conversely, the interesting fibers are situated within the stem, making the reduction of its length much less wise for economic reasons; thus, more suitable solutions are required to avoid lodging without penalization of the fiber yield. The understanding of this phenomenon, challenging the biomechanics of flax plants, is the subject of the next section.

### Lodging of Flax: Investigations Toward a Better Lodging Resistance

The lodging phenomenon is of crucial importance, as it can greatly impact the process of flax harvesting together with fiber yields (Vera et al., 2012). Because plants are intertwined and close to the ground (Figure 9), the use of agricultural machines is much more complicated and the harvesting process slowed down. In addition, lodging constrains the plants to high moisture conditions when laid on the soil; this impacts the harvesting process by increasing the mass of the plants covered with mud but also promotes the development of fungal diseases, such as Pasmo caused by *Septoria linicola* (Muir and Westcott, 2003). However, only a few studies focus on the understanding of the lodging of flax, which makes this phenomenon quite complicated to restrain.

For food crops, two main types of lodging are mentioned in literature, namely, stem lodging and root lodging, and occur through interactions between wind, rain, soil, and the plant (Neenan and Spencer-Smith, 1975; Crook and Ennos, 1994; Berry et al., 2000). Root lodging is due to the failure of the root anchorage and it is preferentially undergone over stem lodging for example by rice (Oladokun and Ennos, 2006). However, within the same field, root lodging can also be accompanied by stem lodging (Berry et al., 2004). Regarding the empirical knowledge of flax farmers, root lodging is rare and, therefore, the given review of lodging mechanisms focuses essentially on stem lodging and structural elements relevant to the lodging vulnerability. In these cases, lodging results in stem buckling (Brazier, 1927), when the strength of the stem in the lodged part is not high enough to withstand the bending moment provoked by the stem leverage (induced by the action of the wind, combined or not with rain) (Berry et al., 2004). For barley, the lodging can occur at any point along the stem, but preferentially at mid-high (Berry et al., 2006). This is explained by the high flexibility of barley associated with a rapid reduction of the strength along the stem toward higher positions (Berry et al., 2006). On the other hand, in the example of wheat, lodging takes place close to the soil surface, in the very basal part of the stem (Berry et al., 2000); this type of lodging is also happening for flax, according to flax farmers. To prevent lodging of wheat, shorter varieties are favorable, as well as strong stemmed plants in the basal part. Thus, the quantity of matter located in the first internode is a crucial factor; in this way, the seedling rate, date of sowing, residual soil nitrogen with reduced inputs of it, and use of plant growth regulators (PGRs) is beneficial for the stems (Berry et al., 2003a,b,c). These parameters were also previously mentioned being advantageous for flax, emphasizing the appropriate parallel between these two crops.

If assimilated to an ideal column, the global buckling of a plant can be approached by considering the Euler column formula (Niklas, 1992). Even though plants are in fact never ideal columns, the Euler column formula provides insights into the relations between variables (being much more complex in actual biological perspectives); it is a reasonable approximation for plant stems, which are free at their top and are anchored at their base by roots. The Euler column formula, for an ideal column anchored at its base and free at its top, is expressed by:

$$P_{crit}=\frac{\pi^{2}EI}{4H^{2}}$$

where *P_crit_* is the critical buckling load, *E* the apparent elastic modulus, *I* the second moment of inertia (being $I=\frac{\pi D^{4}}{64}$ for a circular section), and *H* the height of the column (Niklas, 1992). This equation shows that an increase in stem length or decrease in stem diameter (*I* is proportional to *D*^4^), i.e., an increase of the slenderness ratio, result in reducing the critical buckling load and therefore increase the risk of buckling. The influence of the stem properties is also represented (distinctly) by the apparent elastic modulus *E*. Finally, a stout structure is able to support greater load than a slender one composed of the same material, i.e., a high slenderness ratio would imply an enlarged risk of buckling, setting a potential limit of standing plant structures (McMahon, 1973).

A complementary critical parameter to consider is the critical buckling height. This latter parameter, derived from the Euler column formula, is defined as the maximal height that the plant can reach while remaining stable, given its diameter and material properties (Greenhill, 1881). It is given by the Greenhill equation (also found as Euler–Greenhill formula) (Greenhill, 1881; Niklas, 1998), expressed for a vertical column such as a stem by:

$$H_{crit}=K{\left(\frac{ED^{2}}{\rho }\right)}^{1/3}$$

where *H_crit_* is the critical buckling height, *K* is a proportionality constant, *E* is the apparent elastic modulus of the stem, *D* is the diameter at the base, and ρ is the bulk density of the material (Greenhill, 1881). It highlights that, for given material properties, the decrease of the stem diameter reduces the maximum height that a stem can attain before failure. In addition, the critical buckling height depends on other parameters defining the proportionally constant *K*; these parameters are the distribution of diameters (i.e., the tapering) and the distribution of mass along the stem, as well as the gradients in mechanical properties along the stem (Holbrook and Putz, 1989; Jaouen et al., 2007). Finally, the safety factor can be defined; it is the ratio between the critical buckling height and the actual height of the plant (Niklas, 1993b, 1994).

The exposed formulas confirm the key importance of the flax slenderness as a factor to understand plant instabilities, such as lodging. It also explains the solution of stem length shortening adopted for food crops having increased grain yields. Additionally, these formulas highlight the significance of the material properties. For instance, light-weight materials (i.e., having low bulk density ρ) are preferable over heavyweight ones. In addition, the apparent elastic modulus *E* is confirmed to have a great role toward the resistance to buckling, as slender plants must develop stiffer stems to prevent instabilities. In conclusion, higher density-specific stiffness (E/ρ) are required to decrease the plant vulnerability to buckling (Niklas, 1993a). Nevertheless, the density-specific stiffness precise values, as well as the proportionality constant, are difficult to predict; of course, they differ between plant species (Niklas, 1998), but they also vary as a function of stem development and growth too (Holbrook and Putz, 1989).

As complements to mechanical characterization of the stems, anatomical analyses are of interest to understand lodging. Ford et al. (1979) suggested that solid stems of some wheat cultivars, i.e., filled with pith, have a greater apparent stiffness thus an increased resistance to lodging than cultivars having the hollow stems. This result was demonstrated later as well by Leblicq et al. (2015) through a mechanical analysis of the bending behavior of wheat stems; these authors demonstrated that the bending resistance of crop stems is significantly increased by the presence of a cellular core of lower density than the outer part of the stem. For rice as well, low density tissues comparable with a foam visible in the inner part of the stem are favorable to the stem lodging resistance (Gardiner et al., 2016). In the study performed in 1982, Menoux et al. (1982) investigated the morphological characteristics of flax stems as well as cultural conditions influencing the lodging resistance based on the comparison of several varieties. The authors showed that flax plants are sensible to lodging during the fast growth stage, from 15-cm high to F, but with the most critical time at F (Menoux et al., 1982). These authors also demonstrated that the lodging resistance is linked with the elongation rate during the early growth stage. Indeed, the resistance to lodging is increased for plants having a slow elongation, i.e., exhibiting a short internode length in the basal 18 cm and a low fiber length-to-diameter ratio. Thus, all parameters inducing a slow elongation rate at the beginning of the plant growth is in favor of the lodging resistance. In this way, as nitrogen is known to advantage plant growth and to increase the yield (Easson and Long, 1992; Sultana, 1992), a rather low nitrogen content in soil, as long as providing a good compromise with the fiber yield, is appropriate to decrease the risk of lodging (Dimmock et al., 2005). The use of PGR is also a widespread solution used by farmers to decrease the risk of lodging (Hoffmann, 1992); nevertheless, this practice is not consistent with evolution toward a more environmentally friendly agriculture. In addition, a reasonably low sowing density is suitable to diminish the risk of lodging if not compromising the fiber yield (Easson and Long, 1992), especially due to the linked stem diameter increase, ensuring a better stability of stems. Regarding the link between the stem properties, the fiber content and the lodging stability, Dorst (1953) affirms that breeders could find a favorable combination of lodging resistance (or high stem strength) and fiber content if the two parameters are seemed as independent ones. In 2015, the study of Bourmaud et al. (2015) highlights the role of fibers in the support of the plant. The same year, a correlation between the stiffness of the stem and the stiffness of its fibers was found (Gibaud et al., 2015). However, little is known about the mechanisms of flax lodging, but the development of strategies to avoid the phenomenon requires an understanding of the factors influencing plant vulnerability to lodging.

Finally, based on literature, the phenomenon of flax lodging is expected to be linked to the plant development itself, besides meteorological parameters that also affect this pattern. In fact, the lodging would be greatly related to both elongation and thickening of the cells, more particularly fibers, and the secondary growth of the plant, namely, the xylem part. The changes in fiber as well as the xylem core properties would be of interest, both anatomically and mechanically speaking. Indeed, these processes would define the mechanical stiffness of the stems, by determining the length-to-diameter ratio of flax fibers, as well as their filling rate and stiffness, but also determining the density and thickness of the xylem tissue. Therefore, beyond the fiber yield, the xylem content of new selected varieties would also be a plant property to take into consideration for a more accurate breeding work. Furthermore, concurrently with the fiber content of the stem, the fiber mechanical properties could be a relevant parameter to take as perspectives for breeders to improve the lodging resistance; this parameter is all the more interesting as its optimization would be appropriate for the use of flax fibers in the composite field. Moreover, studies on numerous plant species report an adaptive growth response to external stimuli (Jaffe, 1973; Grace and Russell, 1977; Biddington, 1986); these latter are a possible approach toward the understanding of flax slenderness, stability, and overall biomechanics.

## Influence of Cultural Conditions and of the Environment on the Properties of Flax Stems and Fibers

**FIGURE 10:**
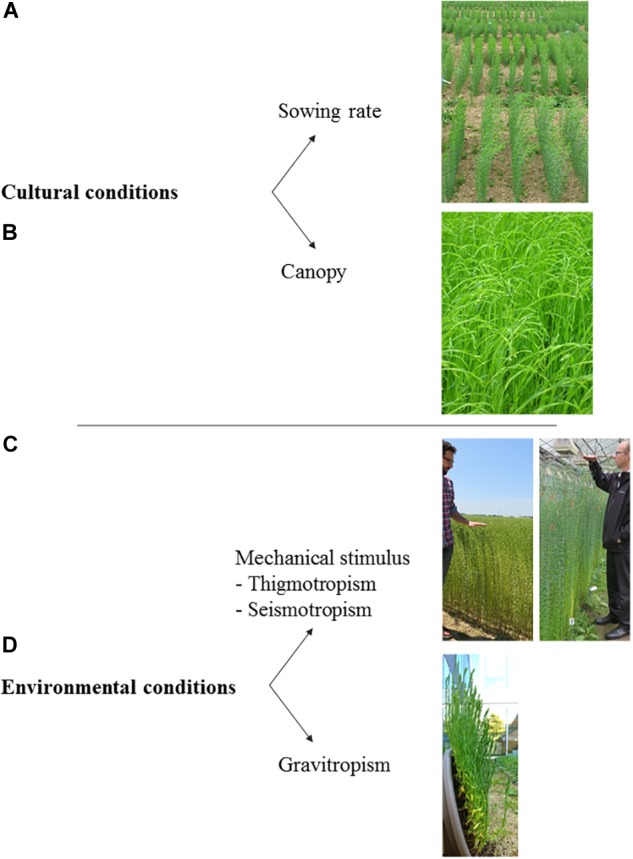
Main notions addressed in this section, with corresponding examples of illustration for flax. **(A)** Sowing density. **(B)** Crop canopy. **(C)** Response to a mechanical stimulus (plants in a field are visible on the left picture whereas they are grown in a greenhouse on the right picture). **(D)** Gravitropic response. The written informed consent was obtained from the two individuals for the publication of this image.

In the present section, the influence of both cultural and environmental conditions on plants is reviewed, with a focus on the case of flax. This implies several notions, which are cited with corresponding illustrations in Figure 10 for a better understanding of resulting plant responses.

### Sowing Rate and Crop Canopy

The number of neighboring trees and their proximity are important parameters for an individual tree growing in a forest; the competition explains changes in height and diameter growth of individual trees (Biging and Dobbertin, 1995; von Oheimb et al., 2011). Similarly, the plant development is very dependent on the sowing rate, the main parameter determining the plant density within a field for industrial crops. Indeed, growing conditions such as light intensity, water and nutrient availability, as well as mechanical challenges strongly vary on whether the plant is growing isolated or within a canopy (Moulia et al., 2011). For example, high flax crop density, most probably due to competition between plant species, can decrease the amount of unwanted weeds within the fields (Stevenson and Wright, 1996); this is an interesting result toward the reduction in the use of herbicides. Reduced line intervals by inducing increased crop density would thus be beneficial in this way. Nowadays, for cereals and flax, the similar in-line seeding technique by means of the same types of crop seed drills is used, controlling the line intervals (Summerscales et al., 2010). The line spacing commonly ranges from 10 to 15 cm, usually 12.5 cm for flax (Figure 10A), with a distance between two consecutive seeds of about 0.5 cm (determined by the speed of the drilling machine), leading to about 0.3 cm between two consecutive stems (Couture et al., 2004a,b; ARVALIS - Institut du végétal, 2014).

First studies in the 80s investigated the influence of the sowing rate on the resulting flax plant growth (Fowler, 1984; Gubbels and Kenaschuk, 1989). For conventional sowing rate of 1800 seeds/m^2^, middle height plant diameter and straw yield are generally around 2.3 mm and 7 kg/ha, respectively (Bourmaud et al.; 2015; Goudenhooft et al.; 2017). Fowler (1984) demonstrated that the sowing rate has a great influence on the plant height (namely, increasing the sowing density decreases the final stem height). Gubbels and Kenaschuk (1989) confirmed this result, if reasonably high sowing rate is used (below 400 seeds/m^2^, the opposite phenomenon is obtained, but this sowing rate is anyway not appropriate for industrial cultivation). The effect of the sowing rate, by influencing the stem height, logically impacts the final scutched fiber length too, i.e., these highest sowing density led to the shortest scutched fiber length (Bourmaud et al., 2016). In addition, increasing the sowing rate decreases the resulting stem diameter; nevertheless, the straw yield and fiber yield are improved, as the increase of the plants per meter square has more impact on the yields than the decrease of the plant height (Easson and Long, 1992; Bourmaud et al., 2016). However, despite decreasing the plant height, high sowing rates negatively influence the lodging stability of the plant, as well as the mechanical properties of flax fibers for greatest sowing rates (Easson and Long, 1992; Bourmaud et al., 2016). Moreover, increasing the sowing density decreases the number of capsules, and consequently seeds, per plant (Casa et al., 1999). Thus, a sowing density providing a trade-off between yields (mainly fiber yield), fiber properties, and lodging resistance is preferred for flax cultivation, namely, a sowing rate of about 1800 seeds/m^2^ is encouraged and commonly used, leading to about 1650–1750 plants/m^2^ thank to high G rates (Easson and Long, 1992; Bourmaud et al., 2016). This results in a relatively homogeneous and dense canopy (Figure 10B). On the other hand, the competition experienced by the plant is more complex than only sowing density dependent. Sowing density, sowing spatial pattern, and G date are indeed interacting factors (Fowler, 1984). In fact, for a conventional sowing density, the time of the emergence of one individual plant related to neighboring ones has a considerable importance on the plant growth; the first emerged plants are greater competitors, and will be the highest all along their development. In addition, they will be even higher, whereas late-emerged plants will be even smaller, than in the case of plants emerged all on the same date (Fowler, 1984). Moreover, the special organization of the plants, such as the diverse line spacing, the different arrangement if along the drilling line or perpendicularly previously mentioned, or as illustrated by the different early-emerged versus lately emerged plants special patterns (Fowler, 1984), will impact on the plant competition and so the plant development. For flax, the best way to regulate the homogeneity of plant emergence is to control the sowing depth of simultaneous stands; namely, a sowing depth of 2 cm is preconized for a homogeneous emergence (O’connor and Gusta, 1994; Couture et al., 2004b). To a lesser extent, the seedbed preparation, such as soil rolling especially for lighter soils (Couture et al., 2004b) or the use of medium conservation tillage to prevent soil erosion (Lafond et al., 1996; Couture et al., 2004a), can be a complement to the use of conventional 2-cm sowing depth.

In the case of a relatively dense and homogeneous canopy, such as a forest or a flax crop field, the interactions between wind and plants (more specifically the whole canopy) are of great interest, particularly as regards lodging induced by wind (Berry et al., 2004). Given the conventional sowing rate used for flax and the sowing recommendations mentioned above, a flax field can hence be assimilated to a dense and uniform continuous canopy structure. The following section shortly reviews the interactions between wind and crop canopies [excluding sparse fields, i.e., the plant spacing is in the range of plant height or more (Raupach et al., 1996), which is not the case for flax] in order to investigate the influence of wind-induced movements on flax lodging.

### Wind-Induced Movements of Crop Canopies

**FIGURE 11:**
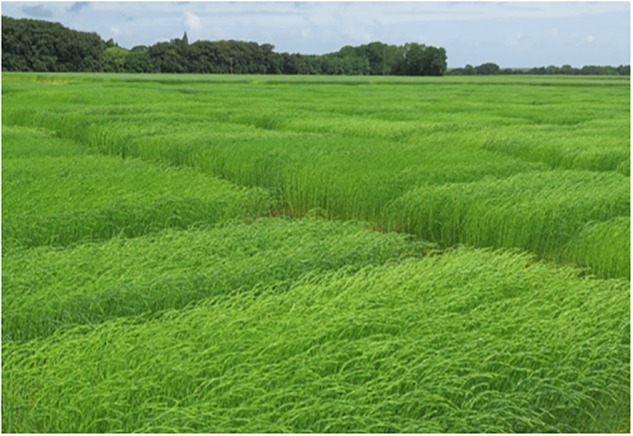
Flax plants moving in the wind in a wave-like movement. The wave is visible as it bends over the plants as it passes.

At first sight, wind is visible as waves over the upper layer of a crop field (Figure 11), as is the case for flax or wheat ones; this wavelike motion of plants was referred to as *honami* first by Inoue (1955) in a study of rice fields. Even though the effects of wind on plants are the subject of many studies on a large range of plant species (Crook and Ennos, 1994; Berry et al., 2003c, 2006), little is known about the specific case of flax. Nevertheless, the interactions between wind and crop canopies are thought to be valid for a flax canopy as well.

Based on literature, plant stems are very lightweight and flexible structures that are typically modeled as isolated elementary oscillating rods (Finnigan, 1979, 2000). These plants assimilated as rods have a natural resonance frequency at which they oscillate (Farquhar et al., 2003). Finally, the plant motion is defined by several plant parameters, namely, its natural resonance frequency, its flexural stiffness and modal mass, a reference damping, and a modal displacement shape (de Langre, 2008). However, within a canopy, the plants are not individualized, but their properties and interactions can be seen as the canopy characteristics; hence, a canopy can be modeled as a discrete set of oscillating identical rods (Farquhar et al., 2003; Doaré et al., 2004; de Langre, 2008). In short, the motion of the canopy is described by the oscillations of a set of stems along a vertical row. In a complementary manner, the average distance between plants is a parameter to consider when modeling the coupling between plants (Py et al., 2006) so the effects of elastic contacts between neighboring crops along a row have been assessed; collisions were found to increase the resonant frequency of individual plants while providing additional support to the stems (Doaré et al., 2004). Crops motions in the canopy induced by wind are then described in literature in relation to wind characteristics. In the absence of plants, the horizontal distribution of wind mean velocity is logarithmic, increasing with the height, and exists as a so-called boundary layer flow (Finnigan, 2000; de Langre, 2008). Conversely, in a canopy, wind that bends over the plants as it passes, occurs as a flow within an inner boundary layer, also called canopy layer; it is then associated with the outer boundary layer flow above the canopy. However, the canopy layer shows great differences regarding turbulence characteristics when compared to the boundary layer, due to the inflection in the wind mean velocity profile, with this inflection becoming even greater over gust events (Finnigan, 2000; de Langre, 2008). The differences between the two flows create a zone of turbulence near the top of a canopy; this zone is called the plane mixing layer and the turbulence is assimilated to Kelvin–Helmholtz instabilities (Raupach et al., 1996). Yet, the impact on flows of the presence of a snap point on plants composing such a canopy has to be investigated. Turbulence emerging in the mixing layer is, nevertheless, not random, with the major contribution of turbulent motions coming from coherent eddies of the scale of the canopy height (Raupach et al., 1996). Indeed, the plants oscillate at their common natural frequency, with a different small phase between adjacent plants when slightly sheltered from the gust, leading to the impression of *honami* waves moving through the field (Finnigan, 1979). When the gust frequency reaches the natural frequency of the plants, a resonant interaction results in a more pronounced waving and stronger *honami* which may lead to crop damage such as lodging (Finnigan, 1979).

This bibliographic synthesis enables to highlight several elements of thought related to the development and stability of flax. The link between stem stiffness, induced movement, and lodging resistance is demonstrated. Thus, measuring the stem stiffness can therefore be a good indicator of lodging resistance, because it directly influences the resonance frequency. In addition, due to the geometry of the seed drills, the distances between the stems are very different depending on the axis considered. In this way, the sowing course could be adapted according to the major wind direction, as is, for example, the case for seaside crops. By standardizing crop density, this would reduce the probability of reaching stem resonance frequency during severe weather events. Finally, interestingly, the presence of a flexible zone between the apex of the plant and the snap point during the growth of a flax stem could modify the turbulence induced by the wind at the top of the canopy and why not create a transitional regime more favorable to the plant stability. Therefore, the presence of the snap point on the natural frequency and the motion of the plants would be interesting parameters to investigate. Moreover, wind-induced motion does not necessarily have a negative impact on yields. It can indeed influence plant growth and biomass allocation as well, phenomena that will be discussed in the following section.

### Tropisms and Plant Responses

Living plants have the ability to respond to a wide range of changes in their environments and they can regulate their patterns of growth in accordance with the stimuli. A growth response involving an active motion due to a stimulus is called a tropism, coming from the Greek word *trepein* meaning “to change direction” (Moulia, 2013); a tropism can be either positive (a response toward the stimulus) or negative (a response away from the stimulus) (Evert and Eichorn, 2013). Major plant tropisms and their consequences on plant characteristics are detailed hereafter.

#### Thigmotropism and Seismotropism

The motion of a plant caused by a mechanical stimulus is often defined by two different tropisms, namely, thigmotropism (from the Greek word *thigma* meaning “touch”) and seismotropism (from the Greek word *seismos* meaning “shaking”) (Hart, 1990). Indeed, a mechanically induced stress can result from either a direct contact such as through passing animals or artificial rubbing of the plant, or from a non-tactile stress such as wind action or artificial ventilation (Biddington, 1986). In the first case, the plant response is called thigmomorphogenesis (Jaffe, 1973) and in the latter, it is often called seismomorphogenesis (Mitchell, 1975); nonetheless, the term thigmomorphogenesis can also be found to describe the plant response to wind (Moulia, 2013; Gardiner et al., 2016). However, this classical categorization according to the contact or non-tactile origin of the mechanical stress is rather confusing, as it can also intertwine both tropisms (for instance, a contact between oscillating neighboring plants can be induced by wind, as previously mentioned in section “Sowing Rate and Crop Canopy”); thus, the growth response induced by a mechanical stimulus would be termed as mechanosensing or mechanoperception (Coutand, 2010; Hamant, 2013; Moulia et al., 2015). Such responses are the subject of many works, but little is known about the special case of flax. Generally speaking, mechanical stimuli have great consequences on the size and shapes of the herbaceous plants and trees, as well as on the mechanical properties of their constitutive tissues. A first example is the widespread reduction in plant height, often accompanied by a greater radial growth of plants submitted to a mechanical stress in many canopies of both herbaceous and wood plants (Figure 10C) (Mitchell, 1975; Biddington, 1986; Niklas, 1996; Moulia and Combes, 2004). This reduction of the plant height is consistent with the reduction of the stem buckling risk and the effective canopy profile to wind, i.e., the plants develop a strategy to minimize the impact on safety (Hamant, 2013; Badel et al., 2015). However, the shortening induced by a mechanical perturbation is not necessarily the adopted strategy, as, for example, wheat plants do not exhibit significant changes in stem height under the influence of wind sway (Crook and Ennos, 1996). Similarly, the increase in plant diameter, even though very common, is not a fundamental rule; indeed, some herbaceous plants have no significant changes in stem diameter despite consistent mechanical stimuli (Goodman and Ennos, 1996; Smith and Ennos, 2003; Paul-Victor and Rowe, 2011), whereas a reduction in diameter of wind-exposed trees can sometimes be observed (Cordero, 1999). Thus, the plant response to a mechanical stimulus can result in changes in the developmental rate, depending on the species, crop density, type of mechanical load, growth stage, growth form (primary or secondary), plant life history, etc., which illustrates the complexity of predicting the plant response (Smith and Ennos, 2003; Paul-Victor and Rowe, 2011; Gardiner et al., 2016). Regarding plant anatomy, changes in constitutive tissue geometry and configuration can also be induced by mechanical stimuli. Changes in tissue geometry can include modification of cell shape and thickness, whereas changes in tissue configuration consist of a modification of the cellulose MFA, arrangement of the cell wall layers, as well as reallocation of biomass in a cross-section. As mentioned previously, at the whole plant morphological scale, there is no fundamental rule to describe a plant response to a mechanical perturbation at the cell scale neither. However, Gardiner et al. (2016) registered the main plant changes induced by wind loading at different scales, for non-woody and wood plants, respectively. Based on this latter review, Table 3 lists the main changes that may be most possibly applicable to flax. In terms of changes in mechanical properties of plants subjected to a mechanical perturbation, Jaffe and Forbes (1993) recorded two types of reactions: increase of the elastic resilience and intensification of the flexural stiffness. These two opposite types of biomechanical responses allow the plants to reach a better resistance to mechanical failure; indeed, increasing the elastic resilience [like it is the case for bean plants *Phaseolus vulgaris* (Jaffe et al., 1984) or *Arabidopsis thaliana* (Paul-Victor and Rowe, 2011)] enables the plants to remain within their elastic limits for greater loads than unstimulated ones, whereas increasing the flexural stiffness [like tomato *Lycopersicon esculentum* stems (Coutand et al., 2000) or *Pinus taeda* pine trees (Telewski and Jaffe, 1986)] avoids exceeding the strain limit.

**Table 3:** Possible acclimation of flax plants as a result of a mechanical stress.

Scale	Flax plants
Stem	Shorter stems Wider stems Changes in plant developmental rate Changes in stiffness Damping
Xylem cells	Increased lignification Increased in xylem tissue density and production Increase in cellulose microfibrillar angle Changes in longitudinal elastic modulus
Fibers	Changes in length, diameter, and wall thickness Increase in cellulose microfibrillar angle Changes in longitudinal elastic modulus Increased strengthening
Phloem cells	Larger phloem vessels

*Adapted from Gardiner et al. (2016).*

Morphological, anatomical, and mechanical changes caused by mechanical perturbations are guided by a trade-off triangle, i.e., plants structure and functions are the natural compromise between the mechanical strength needed to withstand the perturbation, with the conductive efficiency and the resistance to embolism (Badel et al., 2015). Thus, varietal selection, if aiming at optimizing the mechanical properties of flax, could lead to suboptimal performances in one of the two other points of the triangle (Lachenbruch and Mcculloh, 2014; Badel et al., 2015). Interestingly, when plants are subjected to a mechanical stimulus, they become stronger toward mechanical failure by subsequent mechanical perturbations and exhibit an adaptive advantage by becoming less susceptible to injuries than controlled plants (Jaffe and Telewski, 1984). If the plants are able to adapt to a natural mechanical perturbation, they also do so toward artificial solicitations. Finally, if little is known about the impact of a mechanical stimulus on flax, it would be an interesting way to investigate whether it could influence the flax stability toward lodging as well as on mechanical properties of flax fibers in view of optimizing their applications in composite materials.

#### Gravitropism

Another tropism well reported in literature is gravitropism, the plant response to gravity. Actually, gravitropism and thigmomorphogenesis are challenging to disentangle; mechanosensing (better studied in gravitropic responses) is involved in both tropisms and their respective involvement, for example when organs experience bending, is difficultly determined (Coutand, 2010; Gardiner et al., 2016). Gravitropism is very important for agriculture, as it ensures some crops to come back ascending after lodging (Chen et al., 1999). In the case of stem lodging inducing a gravitropic stimulus, the gravitropic response of plants is a stem curvature; at the end of the gravitropic reaction, the stem is generally straight (Coutand, 2010) (Figure 10D).

Gravitropism is attributed to the plant ability to perceive inclination in a first step (Chauvet et al., 2016). This plant aptitude, called gravisensing hereafter, is particularly studied in the case of different herbaceous angiosperms (Chauvet et al., 2016) but also woody ones (Gerttula et al., 2015). As gravisensing is a very complex mechanism studied in several highly detailed articles (Muday, 2001; Coutand et al., 2007; Bastien et al., 2013; Gerttula et al., 2015) but only concise general explanations are given in the present work. In a few words, gravisensing occurs by inclination perception through the sedimentation in the direction of gravity of specialized starch-filled amyloplasts, called statoliths (Sack, 1997). In stems, these latter occur in specialized gravisensing cells named statocytes (Sack, 1997) located in the innermost layer of endodermis cells of the cortex (Fukaki et al., 1998; Hoson et al., 2005; Gerttula et al., 2015), as well as in secondary phloem cells for stems exhibiting a more extensive secondary growth (Gerttula et al., 2015). The sedimentation of statoliths induces a biochemical signal modifying the movements of calcium ions, which then triggers the polar auxin transport and distribution (Hoson et al., 2005; Coutand, 2010). Auxin is the major hormone involved in plant tropism (Morita, 2010), but ethylene (Andersson-Gunnerås et al., 2003) and gibberellic acid (Mauriat and Moritz, 2009) are most probably also involved in the gravisensing signal of woody angiosperms; however, the interactions between these hormones still require further understanding. Changes in the auxin transport and distribution finally result in stem curvature upward. Nevertheless, auxin-related changes differ if considering herbaceous or woody angiosperms, more specifically between stems undergoing primary growth or secondary growth. In fact, changes related to auxin distribution lead to two different types of gravitropic motors inciting stem bending.

In the first case, if the stem undergoes primary growth, the gravitropic motor is the differential elongation growth (Cosgrove, 1997). According to the “Cholodny-Went” hypothesis (Went, 1974), auxin is transported laterally toward the lower side of the stem. It creates an auxin gradient between the upper and lower sides of the stem, inducing an increase elongation growth on the lower side. This gradient results in “pushing” the plant organ upward, i.e., curving of the stem toward a vertical position. In elongating stems, this up-curving is a reaction along the entire growing organ; it starts at the apex, which then becomes straight first by decurving, and the straightening moved downward gradually along the elongating stem (Bastien et al., 2013). Finally, a remaining curvature is visible at the base of the elongating growth zone (Bastien et al., 2013). Actually, gravisensing is accompanied by sensing of local curvature, so-called proprioception, that enables the control of the straightening autotropic response and posture control (Moulia et al., 2006).

In the second case, if the stem undergoes secondary growth, i.e., in stem regions where elongation is finalized but where radial growth happens due to the active cambium, the stem curvature occurs through the asymmetric generation of a specialized xylem tissue called reaction wood (Alméras and Fournier, 2009). In woody angiosperms, the reaction wood is more particularly named tension wood, whereas the xylem formed across from the reaction wood is called opposite wood (Clair et al., 2005; Coutand et al., 2007). Once again, auxin is designated at the origin of the differential cambial growth in woody plants (Kennedy and Farrar, 1965; Forest et al., 2006). Indeed, auxin is distributed toward the cambium and the center of the stem on the upper side of the stem and triggers tension wood formation; conversely, auxin is transported away from the cambium, toward the periphery of the stem on the lower side of the stem and triggers opposite wood formation (Gerttula et al., 2015). Even though it is confirmed that tension wood is capable of generating high tensional stress moving the plant upward (Fisher and Stevenson, 1981; Fang et al., 2008; Mellerowicz and Gorshkova, 2012), the mechanism linking the generation of stress with gravitropism is the subject of many disagreements between authors. In fact, the similarity between opposite wood and normal wood, as well as on the composition and organization of tension wood is clearly accepted. In addition, this latter type of wood, is characterized by a specific G-layer having a high cellulose content, with highly crystalline cellulose microfibrils exhibiting an MFA close to 0° in a free-lignin matrix composed of non-cellulosic polysaccharides and glycosylated proteins (Lafarguette et al., 2004; Mellerowicz and Gorshkova, 2012; Chang et al., 2014). Moreover, it has been demonstrated that the tensile stress in tension wood results from tensions into the cellulose microfibrils of the G-layer (Clair et al., 2006). The origin of tensions in cellulose microfibrils is nevertheless not known with certainty, and different hypotheses were reviewed by Alméras and Clair (2016).

However, even if the generation of tension wood is essentially a characteristic of woody plants, recent studies highlighted its presence in herbaceous stems of *Arabidopsis thaliana* (Wyatt et al., 2010), alfalfa (*Medicago* sativa L.) (Patten et al., 2007), and flax (Ibragimova et al., 2017). These results interestingly confirm the ability of some herbaceous angiosperms to generate tension tissues, somehow analogous to tension wood of woody angiosperms. The study of flax gravitropism is of great interest as this plant naturally exhibits fibers with a G-layer in a normal plant growth, namely the flax primary fibers. In addition, xylem of flax exhibiting G-layer can be produced as a gravitropic response on the upper side of the stem (Ibragimova et al., 2017). This study of Ibragimova et al. (2017) demonstrates that the remaining curvature occurs in a stem part that has already ceased fiber elongation (much below the snap point). However, the fiber thickening, still processing at this level, is impacted by the gravitropic response. As neither cell elongation nor cambium differentiation are involved in the fiber reaction, Ibragimova et al. (2017) suggest that a part of this gravitropic reaction actually occurs within the fiber cell wall itself. Indeed, when comparing fibers of control plants and fibers from the opposite side of the stem, a significant increase of the fiber diameter is obtained on the pulling side (side where reaction wood occurs in the xylem part); in addition, the proportion of the lumen area from fiber area is significantly increased in fibers subjected to gravitropism, on both sides of the stem, with a much greater increase on the pulling side. Last but not least, the shape of the fibers along their length is highly modified, as fibers exhibit “sausage-like” shape with a deposition of callose, a polysaccharide generally present in injured cell walls (Snegireva et al., 2010), in the fiber thinner zones. Finally, the MFA is also impacted by the gravitropic reaction, impacting the characteristics of the G-layer (Ibragimova et al., 2017). Thus, even if little is known about the impact of gravitropism on the mechanical properties of flax fibers and stems, changes in fiber thickening and morphology would most probably influence the fiber mechanical properties and their homogeneity. Even though flax plants are able to recover from lodging through a gravitropic response, stem lodging happening during the early thickening process would jeopardize the mechanical properties of the fibers by impacting this development process. This makes lodging an even more undesired phenomenon, both from the farmer point of view (by decreasing the fiber yield) and the composite manufacturer side (by impacting the fiber properties). If lodging appears closer to FM, fibers would be already well thickened; in this case, a negative direct impact on the fiber properties would be limited, but would still involve greater susceptibility toward diseases and harvesting problems. In this latter situation, the gravitropic response would probably rely essentially on xylem tension wood, demonstrating once more the essential role of this tissue in the flax plant characteristics.

## Conclusion

Flax is one of the oldest plants cultivated by mankind, essentially for the fibers contained in its stem. Flax fibers have always been intended for textile production, including clothing and upholstery. Moreover, a more contemporary application has been advanced over the past decades, namely, the use of flax fibers for composite reinforcement. Growth stages of this plant are quite well described in literature, probably thank to the industrial potential of flax fibers. The processes of fiber development, from initiation to thickening, are the subject of several studies, essentially due to the uncommon properties of flax fibers, morphologically speaking but also due to the remarkable G-layer constituting most of the cell wall. Flax, by being an annual herbaceous plant, providing fibers exhibiting a thick G-layer similar to tension wood as well as a substantial xylem quantity, gathers very promising characteristics to understand the mechanisms of plant responses to a large range of cultural conditions and external stimuli. Nevertheless, it remains difficult to find general average data regarding flax fiber composition, either during plant ontogeny or at FM. This is partially explained by the numerous existing flax varieties and irreproducible meteorological conditions between regions and years inducing a great composition variability, but also due to many protocols followed by authors. In addition, if the meteorological conditions are hardly controllable, the influence of the variety on fiber composition and performances is of greater interest, as it relies on the mastered expertise of varietal selection. This latter has successfully selected flax varieties exhibiting high fiber yields, good disease resistance and a worthy stability toward lodging, while ensuring plant characteristics (height and diameters) remains adapted to existing agricultural machinery and scutching machines. Thus, one can reasonably expect an efficient selection work toward the development of new flax varieties dedicated to technical applications. This innovative approach would come together with studies investigating flax plant adaptation and reinforcement mechanisms in natural or experimental environments, including a complementary interest in the xylem contribution to flax mechanisms. To a larger extent, such investigations would also provide food for thought about the adaptation of flax to current climate changes, including global warming, in order to preserve the cultivation of this outstanding industrial crop.

## Nomenclature

*D*, Stem diameter (m); *E*, Apparent elastic modulus (N/m^2^); *GDD_n_*, Cumulative growing degree-day on day *n* (°C); *H*, Height of the column (m); *H_crit_*, Critical buckling height (m); *I*, Second moment of inertia (m^4^); *K*, Proportionality constant; *P_crit_*, Critical buckling load (N); ρ, Bulk density of the material (N m^-3^); *T_base_*, Base temperature being 5°C for flax (°C); *T_max,i_*, Maximal daily temperature (with *i* equals to 1 on the day of sowing) (°C); *T_min,I_*, Minimal daily temperature on the day *i* (with *i* equals to 1 on the day of sowing) (°C).

## Data Availability

No datasets were generated for this study.

## Author Contributions

AB and CB conceived the review topic. CG organized and wrote the manuscript. AB and CB reviewed the manuscript. All authors approved the manuscript.

## Conflict of Interest Statement

The authors declare that the research was conducted in the absence of any commercial or financial relationships that could be construed as a potential conflict of interest.

## Abbreviations

AFM: atomic force microscopy
F: flowering
F_i_: filial generation number I
FM: fiber maturity
G: germination
G-layer: gelatinous layer
Gn-layer: newly deposited gelatinous layer
L: lumen
MFA: microfibrillar angle
ML: middle lamellae
P_i_: parent number I
PW: primary cell wall
RG I: nascent rhamnogalacturonan
S: senescence
S1: first layer of the secondary wall
S2: second layer of the secondary wall
S3: third layer of the secondary wall
SF: seed formation
SM: seed maturity
SW: secondary wall
THC: delta-9-tetrahydrocannabinol
VS: vegetative stage
